# Cancer Chemotherapy and Chemiluminescence Detection of Reactive Oxygen Species in Human Semen

**DOI:** 10.3390/antiox8100449

**Published:** 2019-10-01

**Authors:** Teppei Takeshima, Shinnosuke Kuroda, Yasushi Yumura

**Affiliations:** Department of Urology, Reproduction Center, Yokohama City University Medical Center, Yokohama 232-0024, Japanyumura@yokohama-cu.ac.jp (Y.Y.)

**Keywords:** reactive oxygen species, oxidative stress, sperm, cancer chemotherapy, antioxidant therapy

## Abstract

Advanced treatments have improved the prognosis of cancer survivors. Anticancer drugs generate large amounts of cellular reactive oxygen species (ROS), but their direct effects on sperm ROS production are unclear. We examined 64 semen samples of men who had received cancer chemotherapy, 467 semen samples of men consulting for idiopathic infertility, and 402 semen samples of partners of female patients as a control group. ROS production was calculated as the integrated chemiluminescence between 0 and 200 seconds after the addition of luminol to unwashed semen. We found that their ROS-positive rate of semen samples in the chemotherapy group was significantly higher than that in the control group. We compared the sperm parameters (concentration and motility) and the ROS production levels between chemotherapy subgroups and one of the remaining subgroups with positive ROS, and we found that only sperm motility was significantly lower in the samples in the postchemotherapy subgroup than in the idiopathic infertility subgroup, and that both sperm parameters were significantly lower in those from postchemotherapy subgroup than in the control subgroup. The ROS production level per million spermatozoa in the postchemotherapy subgroup was significantly higher than that in the control subgroup. Additionally, we compared variables, such as age, sperm features, and the duration from the end of the treatment to the first consultation between ROS-positive and ROS-negative subgroups in samples from men in the postchemotherapy group, but we found no significant differences. Of the men in the postchemotherapy group, three underwent a long-term antioxidant therapy, and all of them had low ROS semen levels after that. In conclusion, the production of ROS in semen detected by chemiluminescence from men who undergo cancer chemotherapy is similar to that of men with idiopathic infertility, and long-term oral antioxidant therapy may reduce the amount of ROS in the semen.

## 1. Introduction

Recently, advances in the treatment modalities for cancer have improved the prognosis of cancer survivors. The five-year survival rate for most types of cancer now exceeds 70% in Japan [1], and the disease has become curable or even chronic for some men. The ability to have children after cancer treatment is a major concern, especially for patients with cancer in the adolescent or young adult populations. Saito et al. reported that 70% of young patients with cancer wish to have children after cancer chemotherapy [2]. However, cancer treatments may impair the fertile capacity of these patients, and approximately 15–30% of patients with cancer have been reported to remain permanently sterile after treatment [3].

Reactive oxygen species (ROS) act as second messengers in cell signaling, whose low levels are essential for various biological processes in cells [4,5,6]. However, oxidative stress, resulting from the imbalances between excessive ROS and low cellular antioxidant levels, leads to deoxyribonucleic acid (DNA) damage and cellular lipid peroxidation.

It has been demonstrated in some studies that anticancer drugs generate elevated cellular levels of ROS via mitochondrial ROS generation and inhibition of the cellular antioxidant system [7,8]. Arsenic trioxide, alkylating agents, vinca alkaloids, topoisomerase inhibitors, platinum compounds, anthracyclines, and bleomycin have all been reported to induce a loss of mitochondrial potential, disruption of the mitochondrial electron transport chain (ETC), electron leakage, and elevated ROS production [9,10,11]. On the other hand, taxanes and nitrogen mustards inhibit the antioxidant system [8]. Amplification of cellular ROS levels by these mechanisms increases the cancer cell ROS threshold to induce cell death [8]. Thus, these drugs have an anticancer effect, but as far as we know, their effects on sperm ROS have not been reported.

For this study, we measured the seminal ROS levels in patients who had undergone cancer chemotherapy and compared them to the levels in patients with idiopathic infertility. Furthermore, we compared semen parameters and ROS production levels between groups and ROS-positive and -negative samples in the postchemotherapy group.

## 2. Materials and Methods

### 2.1. Subjects

We examined 64 semen samples from 64 patients who had received cancer chemotherapy and from 467 patients with idiopathic infertility who had visited our male infertility clinic at the Reproduction Center, Yokohama City University Medical Center, from February 2011 to February 2017, with the chief complaint of low sperm quality. Moreover, we also examined partners of 402 female patients in our center who had normal sperm quality at the first visit and never visited our male infertility clinic as a control group. We formed a “postchemotherapy group”, an “idiopathic group”, and a “control group” with the semen samples. We excluded patients with azoospermia, varicocele, leukocytospermia, and other organic disorders.

We interviewed all the participants and conducted physical and endocrine examinations (testosterone, luteinizing hormone, follicle stimulating hormone, and estradiol) during their first consultation for ruling out varicoceles, seminal tract obstruction, testicular cancer, and other organic disorders. We also compared the patient characteristics, semen features, and semen ROS status between the samples in the three groups. All patients signed informed consent for participation, and the Institutional Review Board of Yokohama City University Medical Center approved this study design (ethical code: IR2502).

### 2.2. Semen Collection and Assessment of Semen Features

We instructed the men to deposit their semen samples through masturbation at our hospital after 48–120 h of sexual abstinence. We then conducted semen analyses using the Sperm Motility Analyzing System (SMAS^™^; DITECT, Tokyo, Japan), a computer-assisted semen analyzer, at 37 °C after 30 min of complete liquefaction. The semen volume (mL), sperm concentration (×10^6^/mL), and sperm motility (%) were measured in accordance with the criteria of the World Health Organization standards of 2010, of which lower reference limits were 1.5 mL for semen volume, 15 million per mL for sperm concentration, and 40% for sperm motility. The reference number of leukocytes was < 10^6^ per mL.

### 2.3. ROS Measurement

We used the Monolight 3010 Luminometer^™^ (BD Biosciences Pharmingen, San Diego, CA, USA) to measure the ROS production levels in unwashed semen while performing the routine semen analysis. We also measured the integrated chemiluminescence between 0 and 200 s before adding luminol to record the chemiluminescence of the samples. Then, we added 40 μL of 100 mM luminol (5-amino-2,3-dihydro-1,4-phthalazinedione) to 500 μL of unwashed semen to obtain the chemiluminescence of the samples expressed as relative light units (RLUs). Then, we calculated the integrated chemiluminescence as the difference between the values, before and after the addition of luminol to the semen samples.

**Figure 1:**
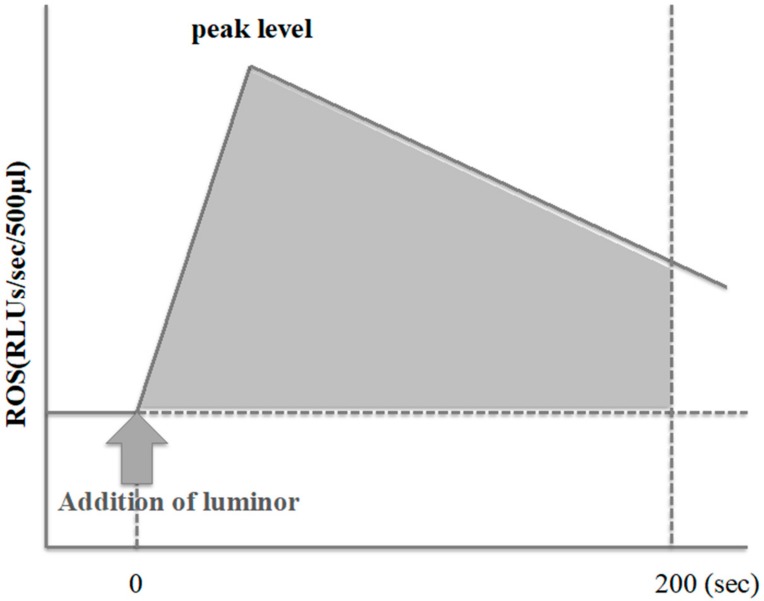
Measurement of reactive oxygen species (ROS) by chemiluminescence method. ROS production in the present study was calculated as the integrated chemiluminescence between 0 and 200 sec after the addition of luminol (5-amino-2,3-dihydro-1,4-phtalazinedione) to unwashed semen after baseline subtraction (expressed as relative light units (RLUs)/s/200 sec).

We considered samples as positive for ROS production if the integrated chemiluminescence was over 4332.4 RLUs/200 s [12] (Figure 1). We then divided the data from the semen samples of the postchemotherapy and control groups into ROS-positive and ROS-negative subgroups.

### 2.4. Statistical Analysis

All statistical analyses were performed using the JMP^®^ Pro 12 software (SAS Institute, Cary, NC, USA). All data are reported as the means ± standard deviation. Group differences (postchemotherapy versus idiopathic groups, postchemotherapy versus control groups, and ROS-positive versus ROS-negative subgroups in the postchemotherapy group) were evaluated using unpaired *t*-tests (parametric variant). We compared the ROS-positive rate between the postchemotherapy and idiopathic groups and the postchemotherapy and control groups using the chi-squared test. *p* values < 0.05 were considered statistically significant in all cases.

## 3. Results

**Figure 2:**
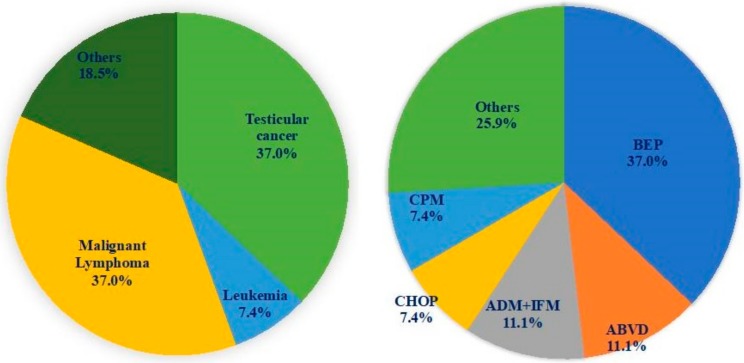
Breakdown of original diseases and chemotherapeutic regimens of those positive for ROS. Testicular cancer and malignant lymphoma were equally frequent. As for the therapeutic regimen, BEP was the most frequent. BEP: Bleomycin, etoposide, and cisplatin, ABVD: Doxorubicin, bleomycin, vinblastine, and dacarbazine, ADM: Doxorubicin, IFM: Ifosfamide, CHOP: Cyclophosphamide, doxorubicin, vincristine, and prednisone, CPM: Cyclophosphamide.

Table 1 shows the characteristics of patients in the three groups. The age at the first consultation was significantly lower in the postchemotherapy group than in the idiopathic and control groups. According to the sperm parameters at the consultation, both sperm concentration and motility were significantly lower in the postchemotherapy group than in the idiopathic infertility and control group, whereas, the ROS-positive rates between the samples from the postchemotherapy and idiopathic infertility groups were similar (42.2% versus 34.4%, *p* = 0.226), that from the control group was significantly lower than that from the postchemotherapy group (20.4% versus 42.2%, *p* < 0.001). Figure 2 shows the breakdown of original diseases and chemotherapeutic regimens of those positive for ROS. Testicular cancer and malignant lymphoma were equally frequent; and as for the therapeutic regimen, BEP (bleomycin, etoposide, and cisplatin) therapy was the most frequently used regimen.

Subsequently, we compared sperm parameters (concentration and motility), total ROS production levels and ROS production levels per one million spermatozoa between postchemotherapy and the other groups positive for ROS (Table 2). Only sperm motility was significantly lower in the samples from the postchemotherapy group than in those from the idiopathic group, but both sperm concentration and motility were significantly lower in those from the postchemotherapy group than in those from the control group positive for ROS. In the control group, sperm parameters were within the normal range at the first visit, but the sperm motility was unexpectedly lower. The total ROS production level in the postchemotherapy group was slightly higher than those in the other groups, but the difference was not statistically significant. As for the ROS production level per one million spermatozoa, no significant difference was seen between the chemotherapy and idiopathic group, but that for the chemotherapy group was significantly higher than for the control group. Additionally, our comparison of the age, sperm features, and the duration from the end of the treatment to the first consultation between ROS-positive and ROS-negative samples in the postchemotherapy group yielded no significant differences between the two groups (Table 3).

Of the patients in the postchemotherapy group, three underwent a long-term antioxidant therapy (combined vitamin C and E supplementation), and all of their semen samples had diminished ROS levels (from 129,745.5 ± 5,383.8 RLUs at the first visit to 15,144.3 ± 10,473.9 RLUs). The mean period from the end of the chemotherapy treatment to the first visit was 20 months, and the mean follow-up duration was 20 months.

## 4. Discussion

Excessive ROS generation in the semen has been observed in approximately 30–40% of men with infertility [12,13], and this unbalance is known to cause lipid peroxidation of the membrane polyunsaturated fatty acids (particularly of docosahexaenoic acids) and to impair sperm mitochondrial respiration, leading to single- or double-stranded DNA fragmentation [14,15]. Lipid peroxidation of sperm cellular membranes causes a loss of membrane fluidity and integrity, which are required for sperm–oocyte fusion [16]. In addition, DNA fragmentation of sperm has adverse effects on embryo development, blastulation, implantation, and pregnancy [17,18]. There have been many reports on the negative effect of ROS on sperm motion parameters [13] and fertile capacity [19,20].

It has been demonstrated in various studies that anticancer drugs produce cellular ROS mainly by two mechanisms: Inducing mitochondrial ROS generation and impairing the cellular antioxidant system [8]. Arsenic trioxide, which was used for leukemia, has been reported to cause a loss of mitochondrial membrane potential and to inhibit complexes I and II, leading to the disruption of the mitochondrial ETC, to electron leakage, and to an elevated ROS production [9,10]. Similarly, alkylating agents, vinca alkaloids, topoisomerase inhibitors, platinum compounds, anthracyclines, and bleomycin generate ROS by the same mechanism [8]. On the other hand, imexon binds to thiols, such as glutathione and causes the suppression of cellular glutathione and accumulation of cellular ROS [21]. Similarly, taxanes and nitrogen mustards inhibit the antioxidant system. In all, these mechanisms amplify the cellular ROS levels and increase the cellular concentrations in cancer cells, resulting in death [8]. Thus, we hypothesized that these anticancer effects might also affect the ROS levels in semen. Anticancer drugs can cross the blood–testicular barrier and impair spermatogenesis. As a result, excessive ROS production in immature sperm may lead to DNA damage. In some studies, there have reported on the effect of BEP therapy for testicular germ cell tumors on sperm DNA integrity [22]. BEP therapy causes single- or double-stranded DNA breaks for more than two years after treatment withdrawal [22]. This may be partly explained by the fact that platinum analogs, such as cisplatin, form cross-links with single- or double-stranded DNA in the sperm, which result in sperm DNA fragmentation and apoptosis [23]. Another mechanism of sperm DNA fragmentation may occur by the accumulation of ROS generated by immature spermatozoa as a result of impaired spermatogenesis, due to cancer chemotherapy. On the contrary, Smit et al. reported that DNA fragmentation index was higher in patients treated with radiotherapy compared with those treated with chemotherapy [24]. In our study, BEP therapy was the most frequently used regimen in men of the ROS-positive group. As mentioned, platinum compounds and bleomycin may disrupt the mitochondrial ETC, leading to the amplification of cellular ROS and subsequent cellular DNA fragmentation [8]. Similar events are thought to occur in sperm cells. Recently, electron spin resonance spectroscopy applying a spin trapping procedure that can measure the oxidative stress, lipid peroxidation, and DNA damage and repair simultaneously has been reported [25].

The total ROS levels were almost equivalent between the samples in the postchemotherapy group, those in the idiopathic group, and the control group. However, produced ROS per unit spermatozoa was significantly higher in the chemotherapy group than the control group. The principal sources of endogenous ROS in semen are immature spermatozoa and seminal leukocytes. In this study, samples with leukocytospermia were excluded. Therefore, in the chemotherapy group, ROS productivity of individual sperm was significantly high. This suggests that defects of spermatogenesis, because of the cancer chemotherapy may result in the accumulation of immature spermatozoa in semen, which produce high levels of ROS. In order to minimize the detrimental effect of ROS on the fertile capacity, an oral antioxidant is considered as an option. According to systematic reviews in the Cochrane database, 48 randomized controlled trials (RCTs) have compared single and combined antioxidants with placebo in a population of 4179 men with infertility [26]. The results suggested that the oral antioxidant therapy may increase clinical pregnancy rates (odds ratio [OR]: 3.43, *p* < 0.0001, seven RCTs, 522 men) and live birth rates (OR: 4.21, *p* < 0.0001, four RCTs, 277 men) [26]. Despite the small number of cases, our results also suggest that long-term antioxidant therapy (combined vitamin C and vitamin E) reduces ROS levels regardless of the period after the cancer treatment and that the oxidative stress is reversible.

The main limitation of this study was the inability to compare ROS levels in semen before and after the administration of cancer chemotherapy, because the semen collected before chemotherapy was all cryopreserved for fertility preservation in consideration of the risk of developing azoospermia permanently after the treatment. Therefore, we cannot be sure that the semen features reflect the cancer chemotherapy effects on ROS in semen.

Another limitation has to do with the inability to evaluate ROS levels depending on the types of cancer and regimens used, because the main subjects were patients whose diseases were in remission, due to cancer treatment and who wished to have babies. However, we believe that cancer chemotherapy may generate excessive ROS in sperm cells, depending on the type of anticancer drugs used.

## 5. Conclusions

In conclusion, our results suggest that the ROS production levels in semen from men who underwent cancer chemotherapy are similar to those of men with idiopathic infertility and that long-term oral antioxidant therapy may reduce ROS levels in the semen.

## Figures and Tables

**Table 1:** Characteristics of patients and semen features in the three groups.

Characteristic	Postchemotherapy (A)	Idiopathic (B)	Control (C)	*p*	
				*A–B*	*A–C*
*n*	64	467	402		
Age (years)	34.9 ± 7.46	37.02 ± 7.00	37.40 ± 6.28	0.015	0.004
Sperm concentration (million /mL)	22.10 ± 31.32	34.75 ± 34.98	44.18 ± 28.93	0.003	<0.001
Sperm motility (%)	23.85 ± 20.63	29.75 ± 21.17	26.90 ± 16.08	0.018	0.03
ROS-positive rate (%)	42.2 (27/64)	34.4 (161/467)	20.4 (82/402)	0.226	<0.001

**Table 2:** Sperm parameters and ROS production levels between both groups positive for ROS.

Parameter	Postchemotherapy (A)	Idiopathic (B)	Control (C)	*p*	
				*A–B*	A–C
*n*	27	161	82		
Sperm concentration (million/mL)	28.66 ± 37.19	28.97 ± 31.55	43.48 ± 24.70	0.482	0.010
Sperm motility (%)	19.36 ± 20.89	26.37 ± 19.89	29.03 ± 14.17	0.047	0.004
ROS level (RLUs)	81032.2 ± 139294.0	76906.7 ± 155068.0	79374.3 ± 273599.9	0.448	0.488
ROS level per million spermatozoa (RLUs)	25937.0 ± 62187.3	21389.6 ± 134787.0	6696.7 ± 26468.2	0.432	0.013

**Table 3:** Comparison between ROS-positive and ROS-negative samples in the postchemotherapy group.

Parameter	ROS-positive	ROS-negative	*p*
*n*	27	37	
Age	35.1 ± 7.3	34.9 ± 7.7	0.456
Sperm concentration (million/mL)	28.66 ± 37.19	17.31 ± 25.74	0.077
Sperm motility (%)	19.36 ± 20.89	21.14 ± 20.08	0.069
The period from treatment to the first visit (month)	34.67 ± 52.57	30.07 ± 70.02	0.387
